# Transcriptional Correlates of Tolerance and Lethality in Mice Predict Ebola Virus Disease Patient Outcomes

**DOI:** 10.1016/j.celrep.2020.01.026

**Published:** 2020-02-11

**Authors:** Adam Price, Atsushi Okumura, Elaine Haddock, Friederike Feldmann, Kimberly Meade-White, Pryanka Sharma, Methinee Artami, W. Ian Lipkin, David W. Threadgill, Heinz Feldmann, Angela L. Rasmussen

**Affiliations:** 1 Center for Infection and Immunity, Columbia Mailman School of Public Health, New York, NY 10032, USA; 2 Laboratory of Virology, Rocky Mountain Laboratories, National Institute of Allergy and Infectious Diseases, National Institutes of Health, Hamilton, MT 59840, USA; 3 Rocky Mountain Veterinary Branch, Rocky Mountain Laboratories, National Institute of Allergy and Infectious Diseases, National Institutes of Health, Hamilton, MT 59840, USA; 4 Department of Molecular and Cellular Medicine, Texas A&M University Health Science Center, College Station, TX 77843, USA; 5 Department of Biochemistry and Biophysics, Texas A&M University, College Station, TX 77843, USA; 6 These authors contributed equally; 7 Lead Contact

## Abstract

Host response to infection is a major determinant of disease severity in Ebola virus disease (EVD), but gene expression programs associated with outcome are poorly characterized. Collaborative Cross (CC) mice develop strain-dependent EVD phenotypes of differential severity, ranging from tolerance to lethality. We screen 10 CC lines and identify clinical, virologic, and transcriptomic features that distinguish tolerant from lethal outcomes. Tolerance is associated with tightly regulated induction of immune and inflammatory responses shortly following infection, as well as reduced inflammatory macrophages and increased antigen-presenting cells, B-1 cells, and γδ T cells. Lethal disease is characterized by suppressed early gene expression and reduced lymphocytes, followed by uncontrolled inflammatory signaling, leading to death. We apply machine learning to predict outcomes with 99% accuracy in mice using transcriptomic profiles. This signature predicts outcomes in a cohort of EVD patients from western Africa with 75% accuracy, demonstrating potential clinical utility.

## In Brief

Using a panel of genetically diverse mice, Price et al. define host responses linked to Ebola virus tolerance and identify distinct gene expression programs underlying pathogenesis. The application of these profiles predicts disease outcomes in mice and human patients.

## Graphical Abstract

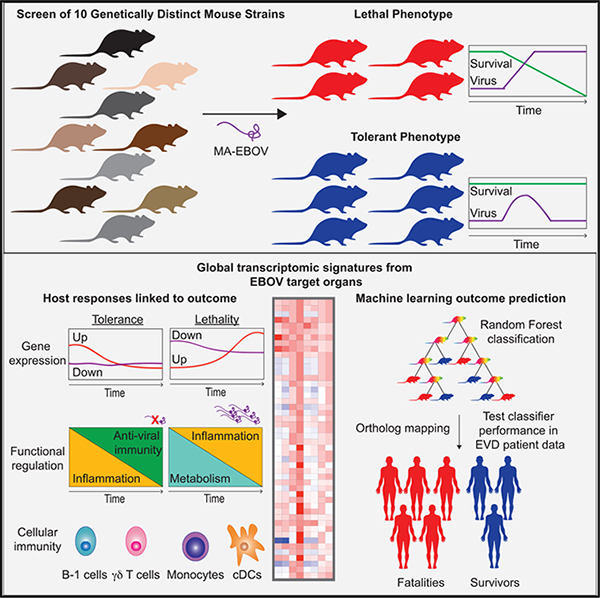

## INTRODUCTION

Ebola virus (EBOV) is an urgent, emerging threat to public health, as outbreaks of Ebola virus disease (EVD) have recently occurred with greater frequency across a wide geographic area. Although some advances have been made with vaccination and experimental therapeutics for EBOV outbreak response and containment, risk factors for susceptibility and severity remain poorly understood. Furthermore, as evidenced by the ongoing outbreak in the Democratic Republic of Congo (DRC), EBOV spread can occur when strategic deployment of vaccines and therapeutics is disrupted (WHO, 2019). Despite the availability and use of experimental countermeasures, the outbreak in the DRC has grown to be the second-largest outbreak after the 2013–2016 western Africa epidemic, which caused more than 28,000 EVD cases (WHO, 2016). These recent outbreaks illustrate the great unmet medical need for new diagnostic and prognostic tools and therapeutic interventions, as well as a more comprehensive understanding of the mechanisms that underlie EVD pathogenesis.

We previously reported a mouse model that recapitulates a broad spectrum of clinical EVD manifestations, ranging in severity from negligible, non-lethal disease to severe, uniformly lethal hemorrhagic disease (Rasmussen et al., 2014). This model uses the Collaborative Cross (CC), an octo-parental panel of reproducible inbred mice that comprise 90% of the genetic diversity across the entire laboratory *Mus musculus* genome (Collaborative Cross Consortium, 2012; Roberts et al., 2007). Two CC lines can be crossed together to create CC recombinant inbred intercrossed (CC-RIX) mice, which have genetic diversity and heterozygosity equal to or greater than comparable human patient populations. In conventional inbred laboratory mice such as BALB/c and C57BL/6, which represent minimal diversity, infection with mouse-adapted EBOV (MA-EBOV) causes lethal disease with no evidence of coagulopathy or hemorrhagic syndrome (Bray et al., 2001). The use of CC and CC-RIX mice enables studying the full range of EVD presentations as well as linking key features of EVD pathogenesis to host genetics. Numerous studies have demonstrated that the host response to infection determines disease severity during EBOV infection, in patient populations as well as in numerous model systems (Caballero et al., 2016; Cross et al., 2018; Ebihara et al., 2011; Eisfeld et al., 2017; Garamszegi et al., 2014; Liu et al., 2017; Rasmussen et al., 2014; Reynard et al., 2019; Rubins et al., 2007; Speranza et al., 2018). As genetic background is a substantial determinant of host responses, the CC model is highly useful for interrogating host responses associated with a particular disease outcome or presentation.

In an expanded screen of CC and CC-RIX mice, we observed that approximately one-third of the lines screened show tolerance to MA-EBOV infection, as defined by productive infection, negligible disease, and greater than 25% survival at day 14 post-infection (p.i.). We performed a detailed study of 10 CC lines (6 tolerant, 1 lethal, and 3 lethal with hemorrhagic disease) to characterize clinical and virological characteristics of tolerant lines compared to those susceptible to lethal disease, as well as to study global transcriptomic responses to infection. In this study, we demonstrate the host responses underlying tolerance to EBOV infection, identify key cell types driving the tolerant phenotype, and develop gene expression signatures that can predict outcome early in infection. We also demonstrate the successful application of predictive host signatures developed in mice to a human patient cohort.

## RESULTS

### Differential Clinical and Virological Outcomes in CC Mice

**Figure 1.**
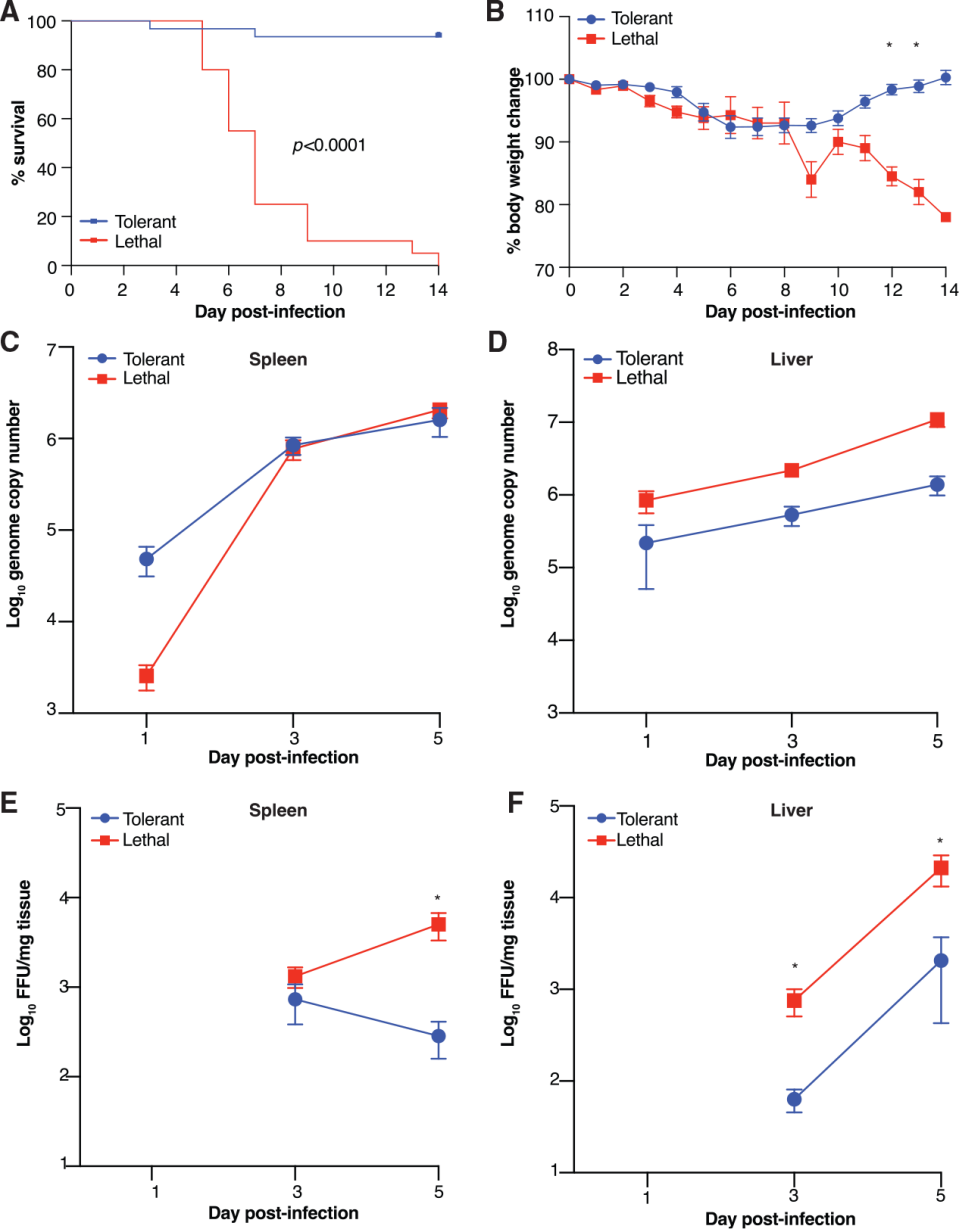
Infection and Disease Course in Tolerant and Lethal EVD (A) Kaplan-Meier survival curve comparing tolerant (blueline; 30 mice) versus lethal (red line; 20 mice) mice over 14 days p.i. Significance determined by the Mantel-Cox logrank test. (B) Body weight percent change from the time of infection (day 0) recorded daily in mice used to assess mortality. * adjusted p < 0.001. (C and D) MA-EBOV RNA genome copies in spleen (C) and liver (D) over a time course from tolerant (15 mice/time point) and lethal (12 mice/time point) outcomes. No viral RNA comparisons met significance criteria (adjusted p < 0.05). (E and F) Viral titers in spleen (E) and liver (F) determined by focus forming assay (FFA) over a time course from tolerant (blue line; 15 mice per time point) and lethal (12 mice/time point) outcomes. * adjusted p < 0.01. Significance was determined using multiple t tests with Bonferroni-Sidak multiple test correction. Viral titer assayed in duplicate. All error bars show SEM.

We screened 50 CC mice from 10 unique CC lines (5 mice/line) for phenotype and outcome (Table S1) after intraperitoneal (i.p.) infection with 100 focus-forming units (FFUs) of MA-EBOV, approximately 10,000 LD_50_ (the dose required to cause lethal disease in 50% of animals) in BALB/c mice. Lethal outcomes were defined by mortality exceeding 75% in a given line. Tolerant outcomes were those with mortality below 25%. We further described disease phenotype by monitoring weight loss and gross observations during necropsy. Our initial screen indicated that six lines were tolerant to infection, and four lines experienced severe, lethal disease (Figures 1A and S1A). All animals lost body weight during the first 6–8 days p.i.; however, tolerant lines fully recovered body weight by the end of the study at day 14 p.i. (Figures 1B and S1B). These are consistent with previous data showing that genetic background determines susceptibility and disease phenotype (Rasmussen et al., 2014), as all animals were infected the same dose of virus.

To assess viral load and confirm that tolerant animals were truly tolerant rather than resistant to infection, we titrated infectious virus and quantified viral RNA in EBOV target organs over a time course. We humanely euthanized animals at serial time points following i.p. infection and collected spleen, liver, and blood. We also collected samples from mock-infected control animals from each CC line. As the primary cellular targets of EBOV infection are macrophages and conventional dendritic cells (cDCs), followed by hepatocytes, we titrated virus in both the spleen and liver by focus-forming assay (FFA) and quantitative real-time reverse transcriptase polymerase chain reaction (qRT-PCR). We observed that in spleen, there were no significant outcome-specific differences in viral RNA throughout infection (Figures 1C and S1C), although there was significantly more infectious virus in animals with lethal disease (Figures 1E and S1E). In liver, there was significantly more viral RNA (Figures 1D and S1D) and infectious virus (Figures 1F and S1F) at days 3 and 5 p.i. No infectious virus was detected at day 1, regardless of outcome. This is consistent with our previous observations that lethal disease correlates with higher viral loads (Rasmussen et al., 2014), as well as with clinical studies that show qRT-PCR cycle threshold (Ct) as a predictor of EVD patient outcome (Crowe et al., 2016; de La Vega et al., 2015; Eisfeld et al., 2017; Kerber et al., 2016; Lanini et al., 2015; Liu et al., 2017; Schieffelin et al., 2014; Shah et al., 2016; Skrable et al., 2017; Yan et al., 2015). Notably, tolerant animals all were productively infected through day 5 p.i., indicating that tolerant animals are susceptible to infection but are capable of controlling it and mitigating disease severity.

### Disease Severity Is Associated with Increased Transcriptional Regulation

Because the only variable in this study is the genetic background of each CC line, we hypothesize that differential host responses drive pathogenesis and outcome. To test this hypothesis, we performed longitudinal RNA sequencing (RNA-seq) on spleen and liver from each of the 10 CC lines in Table S1 infected with 100 FFU MA-EBOV i.p. as described above. Spleen and liver were selected, as each are thought to contribute significantly to EVD pathology. Infected macrophages and cDCs migrate to the secondary lymphoid organs, where they infect other myeloid-lineage antigen-presenting cells and cause bystander apoptosis of lymphocytes. These infected cells then produce large quantities of proinflammatory cytokines. Viremia resulting from infected macrophages and cDCs results in hepatocyte infection and acute hepatitis. Ultimately, hepatocyte death results in liver dysfunction and reduced coagulation factor production. We sought to identify host responses underlying these processes in EVD pathogenesis of varying severity. Global transcriptomic data from infected animals were compared to time-matched, mock-infected controls from each CC line to assess differentially expressed (DE) genes, and data were analyzed using the DEvis package (Price et al., 2019). DE genes were defined as those with fold change > |1.5| relative to the mock-infected controls with an adjusted p < 0.01 (Data S1).

**Figure 2.**
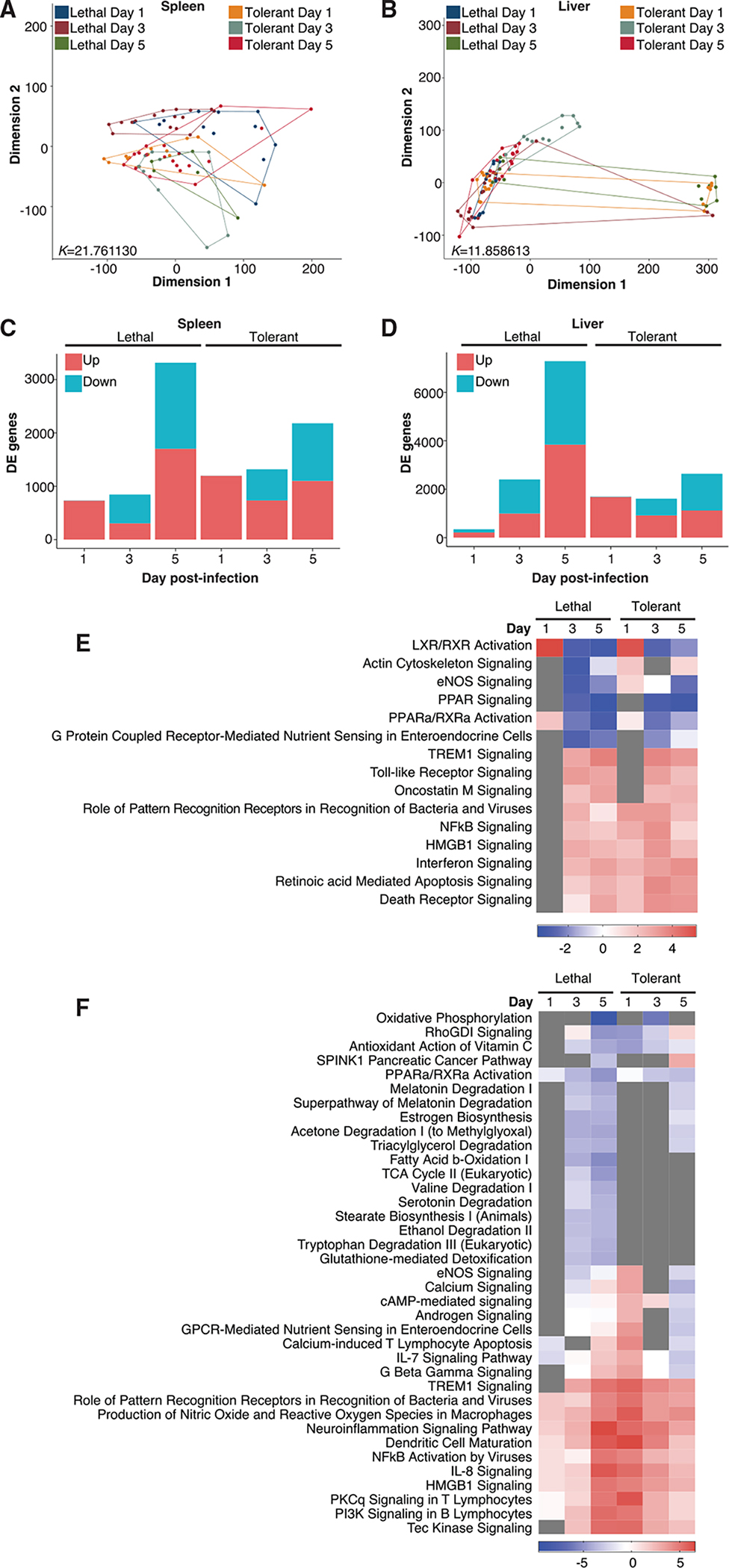
Global EVD Outcome-Dependent Transcriptomic Profiles (A and B) MDS of all differentially expressed (DE) genes in spleen (A) and in liver (B) by outcome and time. K values indicate Kruskal’s stress, a measure of fit and information loss. (C and D) Genes meeting DE criteria (fold change > |1.5|, adjusted p < 0.01) in spleen (C) and liver (D). Blue, downregulation; red, upregulation. (E and F) Pathway enrichment in spleen (E) and liver (F). Enrichment p < 0.01. Transcriptomic data were generated using spleen and liver from mice and analyzed by outcome relative to time-matched mock-infected controls from each line (3 mice/time point/condition/line). Transcriptomic data RNA was obtained from 6 tolerant lines (54 infected mice and 54 controls) and 4 lethal lines (36 infected mice and 36 controls). Each line was sequenced as a single replicate run.

To understand how overall gene expression in tolerance differs from that of lethal EVD over time, we used multi-dimensional scaling (MDS), a dimensionality reduction technique that compresses high-dimensional data such as transcriptomic data and allows visualization of differences between datasets by Euclidean distance on a two-dimensional plane. In both the spleen (Figure 2A) and liver (Figure 2B), we observed that while there was overlap across all outcomes and times p.i., there was also substantial divergence, particularly at later time points. We also analyzed separation using conventional principal-component analysis (PCA) and observed similar overlap at early time points (Figure S3). This is expected when attempting to separate gene expression profiles from genetically diverse individuals such as the different CC lines we used. In both tissues, there was considerably more separation by outcome at day 5 than at day 1, suggesting that distinct clinical outcomes are also more transcriptionally distinguishable across the different CC genetic backgrounds. While DE genes increase over time in both tissues for both outcomes, lethal outcomes are associated with more DE genes than were tolerant outcomes (Figures 2C and 2D), suggesting that tolerance may be associated with more tightly controlled gene expression than in lethal outcomes.

We also used ingenuity pathway analysis (IPA) to analyze the pathway enrichment of DE genes for each outcome over time to better understand functional importance (Data S1). In spleen, we observed that in tolerant animals, pathways associated with antiviral function—including pattern recognition receptors (PRRs), type I interferon (IFN), and NFκB signaling—were all significantly enriched and activated at day 1 p.i. in tolerant animals (Figure 2E). In lethal animals at day 1 p.i., however, these antiviral pathways were not enriched at all, suggesting that the antiviral response is blocked early after infection. The early antiviral gene expression program in secondary lymphoid organs may be critical to the effective regulation of host responses that control infection and clinical disease severity.

In liver, we observed a similar pattern (Figure 2F). While animals with a lethal outcome did upregulate antiviral and inflammatory mediators—such as PRRs, IFN signaling, phagocyte differentiation lymphocyte activation, interleukin (IL)-8 signaling, and NFκB signaling—these pathways became increasingly activated over time, indicating virus amplification and a time-dependent loss of regulatory control. However, in tolerant animals, these pathways were strongly activated at day 1 p.i. and grew progressively weaker, suggesting that a potent early response may result in control of virus replication and host inflammatory responses. We also observed a number of liver-specific differences between outcomes. In animals with lethal EVD, we observed a strong suppression of metabolic pathways and pathways associated with sex hormone and neurotransmitter synthesis beginning at day 3 p.i. Many of these pathways were either not enriched or only mildly inhibited in tolerant animals, suggesting metabolic dysfunction that may contribute to reduced energy production, oxidative stress, and liver injury in severe EVD. These data show the critical importance of early transcriptional responses to EBOV in determining outcome.

Because microRNA (miRNA) are fundamentally related to transcriptional regulation, we also obtained global miRNA-seq data and identified DE miRNA signatures relative to time-matched mock-infected controls (Figure S2; Data S1). We observed no DE miRNA in spleen at day 1 p.i. regardless of outcome, suggesting that miRNA-mediated regulation of gene expression does not occur acutely. In both spleen and liver, DE miRNA expression peaked at day 5 p.i. We used IPA to analyze miRNA function at day 5 p.i.; however, there were insufficient DE miRNA to assess specific pathway enrichment, so our analysis was restricted to more general functional categories (Data S1). In spleen, functional enrichment was notably different based on outcome, with suppression of cell proliferation and migration associated with tolerance but not with lethal outcomes. In liver, we observed similar pathways but did not detect outcome-specific differences (Figure S2). Distinct functional enrichment in the spleen may suggest increased regulation of immune cell growth and differentiation in tolerant animals; however, the enriched functions are based on data derived from immortalized cancer cell lines and may not reflect the *in vivo* function of miRNA during EVD.

### Transcriptional Regulatory Networks Are Linked to Disease Outcome

Because miRNA data alone were insufficient to understand the regulation of biologically important mRNA, we performed an integrative analysis to identify miRNA-mRNA pairs significantly associated with outcome. miRNA that were found to be uniquely DE in either lethal or tolerant animals were selected, and miRWalk (Dweep et al., 2014) was used to identify experimentally validated miRNA-mRNA interactions. DE analysis was performed in parallel on corresponding mRNA sample data and integrated with results from miRNA analysis. This resulted in 71 miRNA-mRNA pairs in spleen and 260 in liver (Data S1).

We then determined miRNA-mRNA pairs for each outcome based on both DE miRNA and mRNA analysis. We identified significant pairs (fold change > |1.5|; p < 0.05) in this manner at days 3 and 5 p.i. for both lethal and tolerant phenotypes in spleen and liver (Data S1). In spleen, we identified 48 pairs that met these criteria at one time point for one or both EVD outcomes. In liver, we identified 208 pairs. Functional analysis was conducted using IPA Core Analysis and the IPA microRNA target filter. Gene Ontology (GO) analysis using ClueGO (Bindea et al., 2009) was performed to confirm IPA results (Data S1).

**Figure 3.**
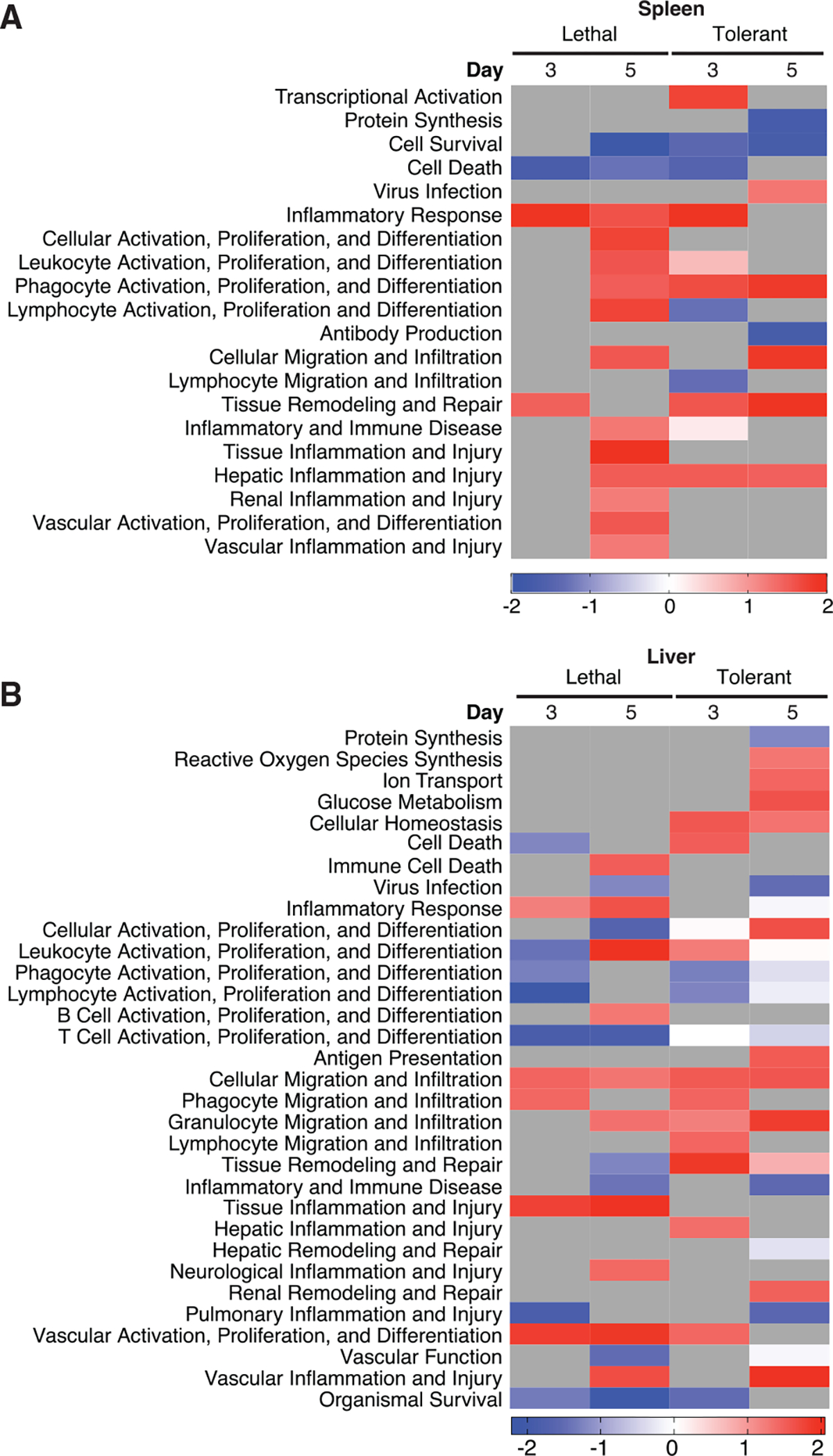
Functional Importance of Transcriptional Regulatory Networks Functional enrichment of miRNA-mRNA pairs in spleen (A) and liver (B) by outcome and time. Enrichment adjusted p < 0.05.

In this analysis, we grouped similar functional categories and excluded all functional categories annotated from cancer, tumor cells, or immortalized cell lines. Spleen transcriptional regulatory networks in lethal outcomes were associated with strong, time-dependent activation of functions associated with inflammatory responses; stimulation and migration of multiple types of immune cells; and vascular inflammation (Figure 3A). This likely reflects unregulated inflammation associated with severe EVD and is consistent with data showing that potent proinflammatory signaling, particularly by phagocytic cells, is a major mechanism underlying pathogenicity (Dutta et al., 2017; Hensley et al., 2002; Lai et al., 2017; McElroy et al., 2019; Menicucci et al., 2017; Mohamadzadeh et al., 2006; Olejnik et al., 2017; Ryabchikova et al., 1996; Wahl-Jensen et al., 2011). In the spleen of tolerant animals, there is also activation of several inflammatory pathways at day 3 p.i.; however, these are not predicted to be activated or are inhibited by day 5. As in Figure 2, splenic inflammatory responses were highly activated at earlier time points in tolerant animals and diminished by day 5 p.i., while in lethal infection, these pathways became more activated over time, suggesting that tolerance is associated with a tightly regulated host response in immune cells, and lethality is characterized by a deficient host response at earlier time points followed by largely unregulated immune activation.

Lethal EVD is likewise associated with increased inflammatory activity at day 5 p.i. in the liver (Figure 3B), as well as pathways associated with vascular activation and injury, and control of vascular barrier function. Although tolerant animals do show increased vascular injury at day 5 p.i., they do not include an accompanying loss of vascular function, nor do they show sustained vascular activation. This observation is consistent with previous studies demonstrating that tolerant CC mice show increased cell death and inflammation of hepatic tissue at day 5 p.i., correlating with peak morbidity; however, this is associated with reduced viral titers compared to animals with lethal disease (Rasmussen et al., 2014).

### Specialized Immune Effector Cells Are Abundant in Tolerant CC Backgrounds

**Figure 4.**
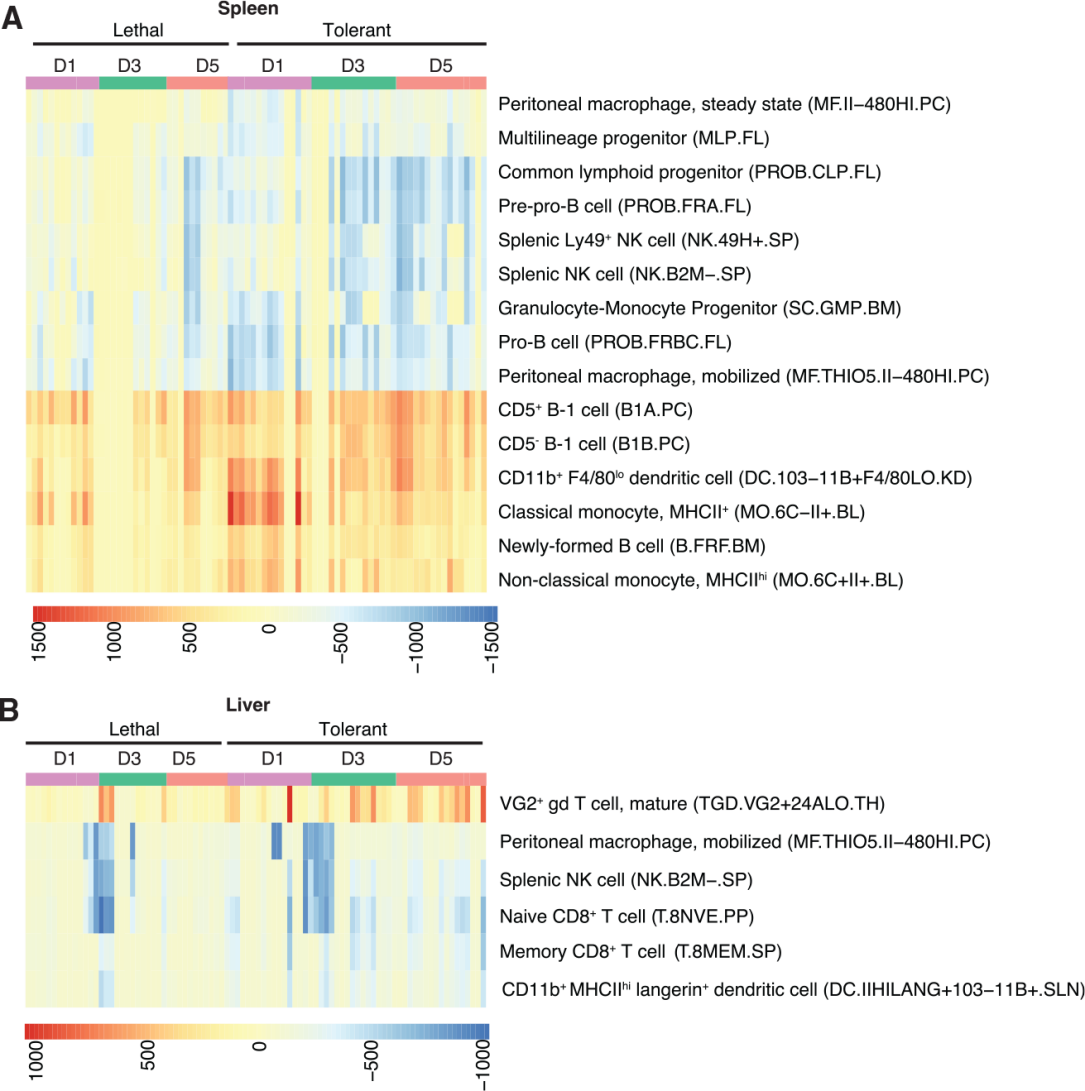
Immune Cell Subsets Linked to EVD Outcome DCQ-derived relative quantities of immune cell subsets in spleen (A) and liver (B). Flow cytometric markers defining each cell type in the ImmGen compendium is shown on the right of each heatmap.

Using the digital cell quantification (DCQ) method, we identified immune cell types of interest (Data S2). The DCQ algorithm determines changes in the relative quantity of specific immune cellular subsets based on the ImmGen compendium of transcriptomic data (Altboum et al., 2014). In tolerant animal spleens, we observed an early and sustained relative decrease in myeloid precursors, B cell precursors, and peritoneal macrophages (Figure 4A). Lethal outcomes were associated with reduced lymphoid progenitors and natural killer (NK) cells only at day 5 and did not show consistent reductions in myeloid-lineage cells associated with inflammation. Tolerant animals also showed early increases in major histocompatibility complex (MHC) class II^+^ monocytes and cDCs, suggesting the rapid mobilization of cells capable of both antigen presentation and regulation of inflammatory signaling. We also observed sustained increases in B-1 cells, suggesting that cells such as cDC and B-1 cells that provide a bridge between innate and adaptive immunity may be responsible for inducing protective, regulated antiviral immunity. Taken together, these data indicate that lethal disease is associated with a loss of inflammatory regulation, consistent with global transcriptomic data (Figure 2E) and integrated transcriptomic network analysis (Figure 3A).

In liver, there were fewer differences between outcomes (Figure 4B). Tolerant animals showed an increase over time in VG2^+^ γδ T cells, which are typically memory cytotoxic cells that produce IL-17A and IFNγ (Ribot et al., 2014). Although some CC lines showed substantial reductions in peritoneal macrophages, NK cells, and CD8+ T cells at day 3 p.i. regardless of outcome, in tolerant animals there appeared to be a sustained reduction in these cell types compared to animals with lethal disease. This indicates that immune cells in the liver may not contribute as substantially to pathogenic host responses as those in the spleen and other secondary lymphoid organs. However, this also suggests that specialized lymphocytes (such as B-1 cells and γδ T cells) are key immune effectors required to mediate protective immune responses in both the spleen and liver.

### Machine Learning Classification Accurately Predicts Outcomes in CC Mice

Genes that are highly differential between outcomes may have predictive abilities that could guide treatment in patients with suspected exposure prior to viremia or clinical illness. We assessed the predictive qualities of such gene sets using machine learning approaches. We used Random Forest (RF), a supervised learning algorithm (Breiman, 2001), to create a model capable of accurately predicting disease outcome based on expression data of a small subset of genes. RFs have been used in a wide range of genomics applications (Chen and Ishwaran, 2012), as well as for predicting clinical outcomes with superior accuracy, compared to other methods (Wu et al., 2003). Furthermore, they have been shown to have high testing accuracy, compared to other machine learning classification methods such as linear discriminant analysis, support vector machines, and neural networks and have been shown to have the best performance among all tree-based classification methods for transcriptomic data analysis (Lee et al., 2005). Finally, RF models allow for the quantitative optimization of parameters, including tree growth and forest size, which can be coupled with bagging, bootstrapping, and random feature selection to minimize overfitting.

**Figure 5.**
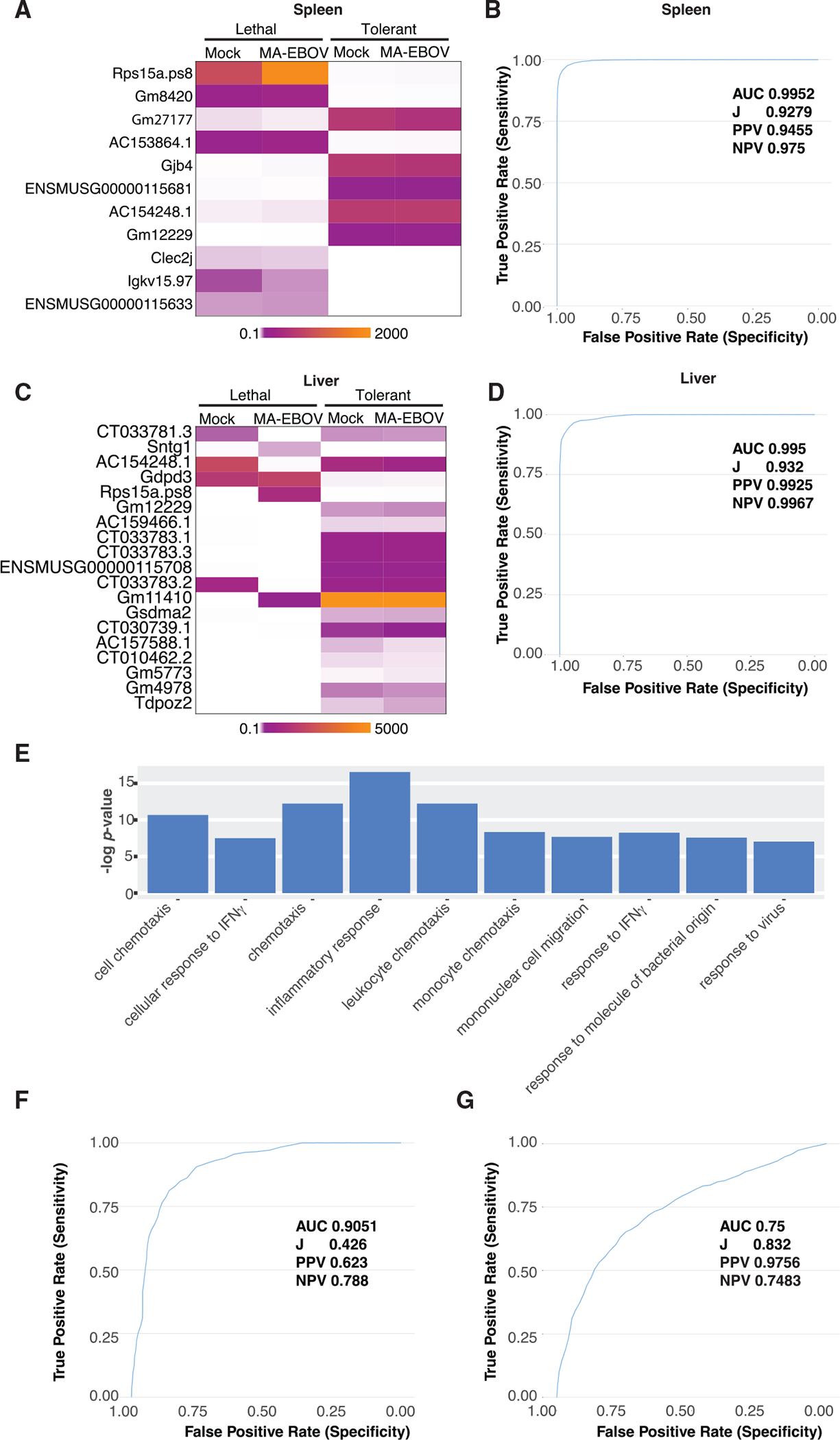
Host Genes Predicting Outcome in Mice and Humans (A–D) Differential gene expression relative to outcome in mouse spleen (A) and liver (C) classifier genes with accompanying ROC curves from spleen (B) and liver (D). (E) XGSA functional enrichment of DE genes from humans and mice. DE genes were defined by fold change relative to controls > |1.5| and Benjamini-Hochberg-adjusted p value. (F) ROC curve of mouse orthologs tested using mouse spleen data. (G) ROC curve of human orthologs tested on human whole-blood data. DE criteria: fold change > |1.5|, adjusted p < 0.01. AUC, area under the curve; J, Youden’s index; PPV, positive predictive value; NPV, negative predictive value.

We identified a subset of DE genes in lethal, relative to tolerant, animals that showed substantial differences in expression (fold change > |1.5| and adjusted p < 0.01; Data S3) between outcome groups at all time points p.i. and trained the model on this subset. The signature was further refined by iterative elimination due to mean decrease in accuracy for each gene, a measure of data importance to accurate classification, resulting in final signatures of 11 genes in spleen (Figure 5A) and 19 genes in liver (Figure 5C). The model was trained on expression data from 40% of randomly selected samples and then tested on the remaining 60% of the samples to determine model performance. Random training and testing were performed 1000 times to calculate sensitivity and specificity. The spleen signature correctly predicted outcomes in 99.52% of tests based on evaluating the sensitivity and the specificity of the assay by measuring the area under a receiver operating characteristic (ROC) curve and calculating Youden’s index (J) (Figure 5B). The liver signature accurately predicted outcomes in 99.5% of tests (Figure 5D). The classifier signature performed well even when accounting for outcome prevalence, with high positive predictive values (PPVs) and negative predictive values (NPVs). PPVs and NPVs are measures of the probability that a test correctly predicts both positive and negative results in the context of outcome prevalence. Each classifier was composed of genes with high variable importance to the model as measured by mean decrease in accuracy. We also observed a low rate of false positives across the 1000 bootstrapped RF runs (Figure S4).

### Predictive Signatures Developed in Mice Predict Human EVD Outcomes with High Accuracy

To assess the translational value of the classifier trained in the CC mouse model of EVD, we tested the accuracy of the predictive signature in mouse spleen using transcriptomic data from EVD patient whole-blood samples obtained in Guinea (Liu et al., 2017). We identified the human orthologs in our classification signature and used the Cross-Species Gene Set Analysis (XGSA) statistical method to identify shared GO functional annotations. This analysis showed that both species share similar EVD-associated processes and that the CC model can be used as a surrogate for human EVD. The XGSA tool was shown to reduce false positives and identify orthologous groups and ontologies better than other available methods (Djordjevic et al., 2016). Using this method, we identified the most significant functional classifications based on DE genes of mice and humans. The results support the hypothesis that similar biological processes are exhibited in both human and mouse EVD, and, therefore, functional insights about infection in one species can be applied to the other. Consistent with our previous analyses (Figures 2 and 3), the 10 most significant functions based on both human and mice DE datasets were related to inflammation, cell migration, and antiviral responses (Figure 5E).

We first tested the cross-species classifier using mouse orthologs. This predicted 90.5% of outcomes correctly (Figure 5F). We then tested the classifier using human EVD patient transcriptomic data, which accurately predicted outcomes in 75% of tests using human whole-blood data (Figure 5G), demonstrating that data from CC mice can correctly predict outcomes across species and tissues regardless of virus titer, clinical disease, or time since exposure. To reflect the disparity in inter-sample correlation likely to be observed in diverse cohorts or caused by suboptimal data quality, we also performed this analysis on the human EVD dataset without filtering for inter-sample correlation. This analysis had minimal effect on classifier performance (Figure S6). The orthologous classifier generally performed well both at all time points in the mouse testing set (Figure S5), although the NPV remains too low to consider this classifier for clinical use.

We used qRT-PCR to confirm expression of 6 out of 14 classifier genes in mouse samples from both this dataset and an independent dataset of 3 tolerant and 3 lethal CC-RIX lines. We observed positive correlations among most of the conditions, with exceptions primarily relating to differential disease outcomes. We also used multiple linear regression to compare the human and mouse RNA-seq data to assess variance by outcome for these 6 genes and observed a high coefficient of determination and a low level of bias. We also showed positive correlations between human and mouse data with similar outcomes (Figure S3; Data S4), demonstrating feasibility for predicting disease outcome across species using this approach.

## DISCUSSION

This study provides insights into the role of host responses in EVD pathogenesis, particularly those associated with tolerance. Prior work has focused on the role of inflammation in severe disease (Cilloniz et al., 2011; Dutta et al., 2017; Ebihara et al., 2011; Garamszegi et al., 2014; Liu et al., 2017; Rasmussen et al., 2014) rather than tolerance. Here, we show that the timing and magnitude of inflammatory gene expression occurrences in EBOV strongly distinguish animals by outcome. These findings greatly expand upon previous observations that an early induction of inflammatory gene expression is associated with asymptomatic EBOV infection (Leroy et al., 2001, 2000) or recovery from EVD (Baize et al., 2002). Notably, tolerance is associated with the induction of key antiviral effectors, including PRR-dependent virus sensing, IFN induction, and NFκB signaling. This suggests that host-directed approaches for reducing EVD severity could enhance the regulation of inflammatory gene expression rather than reduce inflammation itself.

We also observed outcome-specific regulation of metabolic functions, particularly in liver. In lethal EVD, catabolic pathways including fatty acid, amino acid, steroid hormone, and neurotransmitter degradation were inhibited, while they were not affected in tolerant animals. Antioxidant responses such as glutathione synthesis and nuclear factor, erythroid-like 2 (NRF2) were suppressed, while oxidative pathways such as inducible or endothelial nitric oxide synthase (iNOS/eNOS) associated with the production of reactive oxygen species (ROS) were activated. Oxidative stress triggers multiple hematological responses, including aberrant platelet activation, coagulation induction, endothelial inflammation, and increased vascular permeability (Delaney et al., 2016; Fuentes et al., 2018; Ghosh et al., 2015; Pleiner et al., 2003; Scalise et al., 2016; Ulker et al., 2016; Wang et al., 2016). Blockade of oxidative stress responses and ROS production have been implicated in both hemorrhagic fever virus pathogenesis (Aydin et al., 2013; Karadag-Oncel et al., 2014; Narayanan et al., 2014, 2011; Rojas et al., 2007; Soundravally et al., 2014, 2008a, 2008b) and response to lipopolysaccharide (Gandhirajan et al., 2013; Sennoun et al., 2009). As these conditions share many features with severe EVD, it is possible that increased oxidative stress coupled with defective antioxidant responses can directly result in vascular injury and coagulopathy. Enhancing oxidative stress responses should be explored as potential therapies to ameliorate EVD pathology by inhibiting ROS-triggered inflammation, vascular remodeling, and coagulation.

Associating specific immune cell subsets with outcome provides new insight into cellular mechanisms of pathogenesis and protection. During the western African outbreak, fatal EVD cases were associated with severe T-cell dysfunction (Ruibal et al., 2016). Although lymphocytes are not susceptible to EBOV infection, non-antigen-specific activation of effector B and T cells has also been implicated in proinflammatory cytokine production and immune dysfunction (Dahlke et al., 2017; Lubaki et al., 2016; McElroy et al., 2015; Younan et al., 2017). Functional humoral and cellular immunity are inversely correlated with severity, while suppression of adaptive immunity through bystander apoptosis and aberrant lymphocyte responses are characteristic features of lethal EVD (Baize et al., 1999; Bradfute et al., 2008; Warfield et al., 2005). Increased splenic B-1 cells in tolerant mice suggest a role for the early induction of both innate and adaptive immune responses, as B-1 cells are capable of both producing low-affinity IgM and presenting antigen. Unlike macrophages and cDCs, B-1 cells are not susceptible to EBOV infection and therefore can drive antiviral Th1 polarization and immune effector function. Transcriptomic data show that Th1 polarization is highly activated in tolerant animals but inhibited in lethal animals, linking severe EVD to insufficient T-cell immunity. The increased presence of γδ T-cell subsets in the livers of tolerant animals also suggests a protective role for specialized T-cell subsets, likely by acting as cytotoxic effectors that clear infected cells. This is consistent with data linking γδ T-cell effector function to EVD survival (Cimini et al., 2017). Future studies will assess the feasibility of targeting specific immune cell subsets such as B-1 or γδ T cells to ameliorate disease severity, promote virus clearance, and improve vaccine efficacy.

The ability to use mouse models to study EVD pathogenesis may eventually improve early detection and guide patient care during outbreaks. Often, EBOV emerges in regions with limited health care infrastructure and resources. In these settings, highly sensitive diagnostic or prognostic methods that can be adapted to common laboratory equipment such as qRT-PCR thermal cyclers could improve EVD patient outcome overall by diagnosing patients prior to their becoming febrile or symptomatic and guiding subsequent treatment approaches. This is relevant to a recent trial in the DRC indicating that two experimental drugs given individually can significantly reduce mortality, particularly if used to treat patients early (Mulangu et al., 2019). The CC model may be used to study outcomes in the context of different treatments or supportive care protocols without a dependence on limited clinical samples from EVD patients. This could augment efforts to personalize treatment regimens and approaches to clinical care. Even if such signatures are not developed for clinical use, associating transcriptomic profiles with treatment outcome may provide insight into therapeutic mechanisms of action and host correlates of efficacy.

Currently, EVD diagnosis is performed after patients become symptomatic and present for clinical care. Although viral load, as measured by Ct, generally correlates with clinical outcome (Crowe et al., 2016; de La Vega et al., 2015; Eisfeld et al., 2017; Kerber et al., 2016; Lanini et al., 2015; Schieffelin et al., 2014; Shah et al., 2016; Skrable et al., 2017; Yan et al., 2015), prior analysis of a cohort of EVD patients showed that host response signatures were more accurately predictive than Ct (Liu et al., 2017). Our study complements that approach and allows for expanded data collection in the well-defined, diverse CC cohort. Here, we showed that transcriptional biomarkers can predict disease severity in a mouse model and that this can be applied to patient data. Eventually, a similar approach with a larger dataset could lead to the development of clinical assays that could complement existing diagnostic tests. A host-directed multiplex qRT-PCR assay compatible with existing diagnostic technology could provide advantages over Ct alone by providing information to guide patient care. Although the classification profile described in this study is not presently suitable for clinical use as a diagnostic or prognostic assay, it establishes the utility of using the CC model for the development of such signatures without requiring samples from large patient cohorts. Correlated expression in both mouse and human samples (Figure S3) indicates that such biomarker panels may be developed using the CC resource in the future. The CC model’s well-defined genomes will allow us to link host responses associated with disease to genetic features, creating the possibility of genetic susceptibility testing for at-risk populations in the future.

Although predictive power for human EVD outcome using mouse-derived data (Figure 5G) is reduced compared to mice, it is comparable to that shown in models trained within the same human testing set in outcome prediction and outperforms correlation with Ct (Liu et al., 2017), as well as to that reported for biomarker panels developed for other acute viral infections (Parnell et al., 2012; Suarez et al., 2015; Sweeney et al., 2016; Tsalik et al., 2016). We anticipate that future efforts incorporating transcriptomic data from other CC genomes to increase the overall size and diversity of the host response pool will improve predictive performance in human EVD cohorts. Increasing the size and diversity of the CC dataset is essential to future efforts to develop classifiers with translational utility.

While this study focused on tissues that drive EVD pathogenesis, future work should also examine transcriptomic data obtained from CC mouse peripheral blood, which are more comparable to clinically relevant human samples. Proposed biomarker profiles must also be validated in other large EVD transcriptomic datasets, ideally from humans and non-human primates (NHPs) with differential outcomes. Although human EVD transcriptional data are difficult to obtain, the limited data that exist can be supplemented by incorporating additional data from other CC backgrounds and other animal species. Transcriptomic data from NHP studies currently in the public domain were obtained from studies using uniformly lethal doses of EBOV and do not enable testing predictors of tolerance. As more data become available from both EVD patients and animal models, we plan to continue refining these signatures. We also propose assessing classifiers in models of more physiologically relevant mucosal exposure routes. We propose testing classifiers for predictive performance in the context of other infectious diseases, particularly those common in western and central Africa, with shared clinical features of EVD, such as other hemorrhagic fever viruses, typhoid, and malaria. Future work to integrate additional datasets in classifier development and refinement should also use alternative classification approaches, including comparison of means, regression-based approaches, hierarchical clustering, and other machine learning algorithms.

We have now demonstrated that in the EVD model, genetic background and downstream host responses play a critical role in determining severity, and we anticipate that these mechanisms of pathogenicity may be applicable to other diseases with similar pathology as well. The CC model of EVD will be instrumental in further investigating host-driven mechanisms of pathogenesis and defining genomic regions relevant to pathogenesis, as well as for the future development of novel host-targeted molecular and cellular tools for clinical use.

## STAR★METHODS

### LEAD CONTACT AND MATERIALS AVAILABILITY

Further information and requests for resources and reagents should be directed to and will be fulfilled by the Lead Contact, Angela Rasmussen (alr2105@cumc.columbia.edu).

This study did not generate unique new reagents.

### EXPERIMENTAL MODEL AND SUBJECT DETAILS

#### Collaborative Cross mice

Six- to eight-week-old male CC mice were purchased from the University of North Carolina (UNC) Systems Genetics Core Facility. CC lines used for this study were: CC011/Unc, CC021/Unc, CC026/Unc, CC041/TauUnc, CC042/GeniUnc, CC043/GeniUnc, CC055/TauUnc, CC057/Unc, CC061/GeniUnc, and CC065/Unc. Male mice only were used for these studies as specified in the scope of work approved by the funding agency. Additional mice were kindly provided by David Threadgill at Texas A&M University (TAMU). All mice obtained from UNC or TAMU were bred and maintained under the oversight of each respective institution’s animal care and use committee (IACUC). Animal experiments in ABSL-4 containment were approved by the Rocky Mountain Laboratories (RML) IACUC and were performed in accordance with guidelines established by the Association for Assessment and Accreditation of Laboratory Animal Care (AAALAC) by certified staff in an AAALAC-accredited facility. As RML is a NIH facility, all protocols also adhere to the NIH Guide for the Care and Use of Laboratory Animals. In addition, protocols were reviewed by the Animal Care and Use Review Office (ACURO) at the United States Army Medical Research and Development Command. Full annotated CC genome sequences can be obtained at the UNC Systems Genetics Core website (http://csbio.unc.edu/CCstatus/index.py). Animals were acclimated to the BSL-4 laboratory environment for at least five days prior to infection. When possible, littermates were matched in experimental and time-matched mock-infected control groups. Mice were infected intraperitoneally with 100 focus-forming units (FFU) MA-EBOV (corresponding to 10,000 times the dose required to cause lethal disease in 50% of BALB/c mice).

#### Virus Stocks

The MA-EBOV stock used was described in our earlier work (Rasmussen et al., 2014) and obtained from the United States Army Medical Research Institute of Infectious Disease (USAMRIID). Briefly, MA-EBOV was amplified in Vero E6 cells according to methods established at USAMRIID (Bray et al., 2001). Virus stocks were sequenced to confirm consistency with the published sequence, GenBank accession #AF499101 (Ebihara et al., 2006). Virus stock was titrated using the focus-forming assay described below.

#### Cell Culture

Vero E6 (ATCC #CRL-1587) is a spontaneously immortalized kidney epithelial cell line derived from an adult female African green monkey (*Chlorocebus sabaeus*, formerly *Cercopithecus aethiops*). Vero E6 cells are widely used for propagating and titrating viruses, including EBOV and MA-EBOV, as they are completely interferon-deficient (Desmyter et al., 1968). Periodically the Vero E6 cell stocks used at RML are tested to confirm absence of mycoplasma contamination. Cultured Vero E6 cells were maintained in Dulbecco’s modified Eagle’s medium (DMEM; ThermoFisher) with 10% heat-inactivated fetal bovine serum and 1% penicillin/streptomycin (ThermoFisher).

### METHOD DETAILS

#### Study Design

We performed a screen of 10 CC lines purchased from the University of North Carolina Systems Genetics Core to assess clinical, virological, hematological, and transcriptomic aspects of EVD phenotype. For each CC line screened, 5 males were used to assess survival and morbidity over a 14-day time course. Parameters were based on our previous observations that MA-EBOV infection causes lethal disease in 60%–70% of CC and CC-RIX lines tested. Assuming the CC population would have a lethal or severe disease phenotype in 65% of animals tested, the appropriate sample size with a 95% confidence interval is calculated to be 5 animals. Mice were humanely euthanized if they met clinical endpoint criteria (≥20% body weight loss, visible hemorrhage, seizure, paralysis, ataxia, extreme lethargy, tachypnea, or dyspnea) or at day 14 post-infection. Animals were excluded from the study if they showed clinical disease prior to infection (such as after shipping and during or after laboratory acclimation). This occurred for only 2 mice from CC065/Unc. Survival and morbidity experiments were repeated to confirm phenotype for each line at least once using CC mice obtained from a different colony at Texas A&M University.

In addition, for each CC line screened, we performed serial euthanasia on groups of 3 mock-infected controls and 3 MA-EBOV-infected animals at days 1, 3, and 5 post-infection to collect samples for virus titer and transcriptomics. CC mice were assigned to groups to ensure that littermates were evenly distributed and matched across experimental and control conditions. Time points were selected based on our previous studies as optimal for observing early, middle, and late stages of disease. Power analysis was performed according to established methods for laboratory animal experiments (Charan and Kantharia, 2013; Festing and Altman, 2002) using OpenEpi software (http://openepi.com/SampleSize/SSPropor.htm). Group size determination for transcriptomic studies is influenced by variation between biological replicates (number of subjects per group) and technical variation (depth of sequencing) (Hart et al., 2013). Because CC mice are highly homozygous within lines and we sequence to a minimum depth of 30 million single-end reads, we determined that our sample size of 3 animals per condition per time point is sufficient to obtain coefficients of variation within the recommended range specified by the binomial negative model used to perform differential expression analysis of transcriptomic data (Love et al., 2014; Price, 2018).

#### Biosafety and Animals

Experiments were conducted in the biosafety level 4 (BSL-4) laboratory at RML using standard operating procedures approved by the RML Institutional Biosafety Committee (IBC). Sample inactivation was performed according to standard operating procedures approved by the IBC for removal of specimens from BSL-4 containment. Six- to eight-week-old male mice were purchased from the University of North Carolina (UNC) Systems Genetics Core Facility. CC lines used for this study were: CC011/Unc, CC021/Unc, CC026/Unc, CC041/TauUnc, CC042/GeniUnc, CC043/GeniUnc, CC055/TauUnc, CC057/Unc, CC061/GeniUnc, and CC065/Unc. Additional mice were kindly provided by David Threadgill at Texas A&M University from the following lines: CC019/TauUnc × CC004/TauUnc, CC004/TauUnc × CC011/Unc, CC041/TauUnc × CC012/GeniUnc, CC059/TauUnc × CC065/Unc, CC030/GeniUnc × CC061/GeniUnc, and CC011/Unc × CC042/GeniUnc. Full annotated CC genome sequences can be obtained at the UNC Systems Genetics Core website (http://csbio.unc.edu/CCstatus/index.py). Animals were acclimated to the BSL-4 laboratory environment for at least five days prior to infection. Mice were infected intraperitoneally with 100 focus-forming units (FFU) MA-EBOV (corresponding to 10,000 times the dose required to cause lethal disease in 50% of BALB/c mice).

#### Survival and Morbidity Experiments

Groups of at least 5 male CC mice from each line were used to assess survival and morbidity. After acclimation to high containment, animals were infected with 100 FFU MA-EBOV by i.p. injection using a total of 200 μL inoculum delivered bilaterally with a 25-gauge needle. Infected animals were monitored over a 14-day time course, including a minimum of twice-daily checks for clinical condition. Mice were humanely euthanized when they met clinical criteria for humane end point euthanasia.

#### Time Course Experiments

Groups of at least 3 male CC mice per line per time point per condition were used to assess longitudinal disease progression at three time points p.i. Infected mice were injected i.p. with 100 FFU MA-EBOV as described for survival experiments. Time-matched mock-infected animals were i.p. injected in the same manner with 200 μL of virus stock buffer (complete DMEM medium) delivered bilaterally. All mice used for serial sacrifice studies were humanely euthanized at days 1, 3, or 5 p.i. by exsanguination via cardiac puncture while under deep anesthesia with inhalational isoflurane, followed by isoflurane overdose. Samples were not collected from animals found dead; in situations where an animal was found dead prior to euthanasia and necropsy, we repeated the experiment in a replicate animal. Gross pathological observations were recorded at necropsy. Necropsy and sample collection were performed immediately following euthanasia. Spleen and liver samples were collected and frozen for subsequent viral titration, or preserved in RNAlater (ThermoFisher) according to the manufacturer’s protocol and subsequently frozen at −80°C in Trizol reagent for downstream RNA extraction.

#### RNA Extraction and Quality Control

Total RNA was isolated from liver and spleen frozen at −80°C in Trizol reagent (ThermoFisher). RNA was extracted using the Direct-zol MiniPrep RNA extraction kit (Zymo Research). RNA quality was assessed using either the Agilent Bioanalyzer 2100 or Agilent TapeStation. RNA was stored at −80°C until use.

#### Virus Titration

Viral RNA was quantified using reverse transcription-polymerase chain reaction (RT-PCR). cDNA was generated using the SuperScript III Platinum One-Step qRT-PCR kit (ThermoFisher Scientific) and samples were run on a Bio-Rad C1000 Touch thermal cycler. Custom primer/probe sets targeting EBOV GP were purchased from IDT: GP-F-5′- GCAGAGCAAGGACTGATACA-3′. GP-R-5′- GTTCGCATCAAACGGAAAAT-3′. GP-Probe-5′-FAM- CAACAGCTTGGCAATCAGTAGGACAT-TAMRA-3′. Copy numbers were determined using a plasmid containing the GP amplicon for the above primer/probe set.

Virus infectivity titers (FFU) were determined by indirect immunofluorescent staining of serially diluted virus stock or organ homogenates from infected animals in triplicate on confluent Vero E6 cells in a 96-well plate. Following adsorption, cells were overlaid with Eagle’s minimal essential medium (MEM) with 3% FBS and 1.5% carboxymethylcellulose. Cells were incubated for 5 days at 37°C, 5%CO_2_. The overlay was removed by washing with phosphate buffered saline (PBS), and cells were fixed in 10% formalin. Plates were treated for 30 min with 0.25% Triton X-100, immunostained with a monoclonal mouse anti-EBOV VP40 antibody (generously provided by Yoshihiro Kawaoka at the University of Wisconsin-Madison) at a 1:10,000 concentration for 2 h, and incubated with FITC-conjugated secondary antibody (MilliporeSigma). Foci were then enumerated using a fluorescence microscope. Infectious titers were not determined on CC042/GeniUnc samples due to insufficient infectious sample available to perform the focus-forming assay.

#### mRNA Sequencing

Total RNA samples were poly-A enriched and underwent library preparation using Illumina TruSeq v2 reagents. Libraries were run on an Illumina HiSeq 4000 short read sequencer at the Icahn School of Medicine at Mt. Sinai Genomics Core Facility. For each sample, we collected a minimum of 30 million single-end 100 base pair (bp) reads.

#### miRNA Sequencing

Total RNA samples were size selected and underwent library preparation using Illumina TruSeq Small RNA kits. Libraries were run on an Illumina HiSeq 4000 short read sequencer at the Icahn School of Medicine at Mt. Sinai Genomics Core Facility. For each sample, we collected a minimum of 5 million single-end 50 bp reads.

#### RNA-seq Data Normalization and Processing

Adapters were trimmed from raw reads using Trimmomatic (Bolger et al., 2014) and RNA-seq data quality were assessed with FastQC (Andrews, 2010). Reads were aligned to the *M. musculus* GRCm38.p6 (GCA_000001635.8) genome using STAR (Dobin et al., 2013) and transcript quantification was be performed using the R-subread package’s featureCounts utility (Liao et al., 2014).

#### Statistical and Differential Expression Analysis

mRNAs and miRNAs were filtered for quality as described above. miRNA expression values were quantified using miRdeep2 and the miRbase database version 22.1 (Rigden and Fernández, 2019). Differential expression (DE) analysis was then performed using the DESeq2 (Love et al., 2014) and DEvis (Price et al., 2019) software packages to identify mRNAs and miRNAs that were differentially expressed relative to mock samples for both lethal and tolerant outcome groups at 1, 3, and 5 days post-infection. Transcripts were determined to be differentially expressed when having an adjusted p value of less than 0.01 and fold change relative to time-matched mocks greater than |1.5|.

#### Functional Analysis

DE mRNA and miRNA lists were each uploaded to Ingenuity Pathway Analysis (IPA) (QIAGEN) and samples that met DE cutoffs (fold change > |1.5|; adjusted p < 0.01) underwent IPA Core Analysis and IPA miRNA Target Filter Analysis. We filtered lists of enriched pathways by the IPA Benjamini-Hochberg-corrected p value (as determined by Fisher’s exact test) and the z-score where calculated. We also built custom networks using IPA’s Molecular Activity Prediction and Upstream Analysis tools. In addition, we conducted analysis using ClueGO to identify functional patterns in integrated miRNA-mRNA data. We conducted functional analysis based on gene ontology to examine functional similarities in DE genes in mice and humans using the XGSA tool (Djordjevic et al., 2016).

#### Transcriptomic Data Integration

miRNAs that were found to be uniquely differentially expressed in either lethal or tolerant conditions, regardless of time point, were selected and miRWalk (Dweep et al., 2014) was used to identify experimentally validated miRNA-mRNA interactions. Differential expression was performed in parallel on corresponding mRNA sample data and data from genes identified as statistically significant in miRNA analysis were extracted and integrated with results from miRNA analysis. Significance and relative fold change values for each miRNA-mRNA pair were calculated for both miRNA and mRNA experiments at all three time points.

Significant miRNA-mRNA pairs were then determined for each phenotype based on differential significance from both miRNA and mRNA analysis. A miRNA-mRNA pair was considered to be a significant regulatory event only when both miRNA analysis and mRNA analysis independently met significance criteria (p ≤ 0.05). Significant pair identification was performed in this manner at each time point for both lethal and tolerant phenotypes, with pairs meeting significance criteria in both lethal and tolerant groups at all time points being filtered as well.

#### Digital Cell Quantification

Relative cell type quantities were determined from count data using DCQ as implemented in the R-package ComICS using default parameters (Altboum et al., 2014). This method combines gene expression data with a mouse immune cell compendium to infer changes in relative quantities of 217 immune cell types. Deconvoluted cell type quantities were sorted and visualized.

#### Machine Learning Classification

To design our random forest model, a panel of genes was identified that discriminated between outcomes based on differential expression in lethal outcomes relative to tolerant outcomes. To determine which genes to use in the model, we selected a subset of DE genes that showed differences in expression between lethal and tolerant outcome groups (fold change < |1.5| and adjusted p < 0.01) and trained an initial model on this subset. The random forest (RF) software package calculates the mean decrease in accuracy for each gene, describing how important each gene’s data was in modeling accurate predictions. Genes that were identified in this way as being less important or detrimental to the model were iteratively eliminated until a set of genes that were all identified as being beneficial to the model was refined. Data from these genes were split randomly into training and test sets and 1000 iterations were performed to measure accuracy of the model. We evaluated model accuracy using ROC curves. We calculated area under the curve (AUC), Youden’s index (J), positive predictive value (PPV), and negative predictive value (NPV). PPV and NPV were calculated using outcome prevalence in this study (lethal 40%, tolerant 60%).

#### Cross-Species Comparisons

We used RNA-seq data from human EVD patient blood samples obtained in Guinea from 2014–2016 and performed DE analysis independently on human and mouse datasets as described above, with the goal of maintaining consistency between the two analyses. Samples with within-group mean correlation of less than 0.9 and fewer than 10% of reads mapping to the human reference genome were excluded and genes with zero transcripts in more than half of their associated group were also filtered from the dataset. Data were normalized using the DESeq2 implementation of the TMM normalization method and variance stabilization was performed. Differential expression was then calculated for both datasets. In the human dataset, convalescent samples were used as the control for comparison for fatal and survival cases. In the mouse dataset, mock samples were used as the control for comparison to lethal and tolerant mouse lines. As no comparable longitudinal data existed for human samples, mouse samples combined into fatal and survival cases regardless of time point. Differentially expressed genes for both datasets were identified as genes with an adjusted p value of less than 0.05 and a log_2_ fold change of 1.5 or greater as compared to controls.

We constructed ortholog maps of DE human and mouse genes with expression profiles unique to lethal or survival outcomes using Ensembl’s biomaRt package (Yates et al., 2016). Pearson’s product-moment correlation test was used to examine independently calculated expression changes relative to control conditions for these DE genes.

#### Validation of Predictive Signatures in Human Data

We used RF to determine if the DE genes identified through homology mapping could produce predictive models that could distinguish between lethal and survival outcomes using the same gene set in both species. The DE genes identified during cross-species homology mapping was first input into model for predicting human Ebola infection outcomes. This list was refined by iteratively removing genes identified by the model to be less important for prediction until a final predictive set consisting of 14 genes was identified. Using these genes, a model was trained to predict lethal or survival outcomes based on expression data. We evaluated model accuracy using ROC curves after 1000 bootstrapped runs when tested in the human data. These same genes were used to train a classifier based on the mouse data for these genes, again evaluating accuracy using ROC curves after 1000 bootstrapped runs. For each ROC curve, we calculated area under the curve (AUC), Youden’s index (J), positive predictive value (PPV), and negative predictive value (NPV) as described in the “Statistical Details” section. PPV and NPV were calculated using outcome prevalence within each model (mouse: lethal 40%; human filtered: lethal 78.57%; human unfiltered: lethal 81.1%).

#### Correlation of qRT-PCR to RNA-seq Data

We used TaqMan Gene Expression primer/probe sets (ThermoFisher Scientific) with TaqMan master mix to measure expression of 6 genes from the 14 gene classifier signature per the manufacturer’s protocol. First-strand cDNA was generated from 50 ng RNA from each sample using the SuperScript III reverse transcription kit (ThermoFisher Scientific) according to the manufacturer’s protocol. All qRT-PCR reactions were performed in duplicate. We used the following FAM/MGB-labeled assays: ANKRD22 (Mm04208511_g1), IL1R2 (Mm00439622_m1), OAS2 (Mm00460961_m1), MX1 (Mm00487796_m1), MX2 (Mm00488995_m1), and FPR2 (Mm00484464_s1) multiplexed with a VIC/TAMRA-labeled 18S rRNA endogenous control assay (catalog #4310893E). All qRT-PCR assays were performed on a Bio-Rad CFX Touch Real-Tme PCR Detection System. Quantitation was performed relative to control (mock) samples and 18S expression using the 2^−ΔΔCt^ method.

### QUANTIFICATION AND STATISTICAL ANALYSIS

#### Statistical details

We have included group sizes, number of replicates, statistical tests, and multiple test corrections applied, and significance criteria in figure legends and in the main text of the manuscript. In general, we used the Mantel-Cox log-rank test to determine significant differences in survival and t tests to determine differences in body weight and viral load. Transcriptomic analyses relied on statistical tests and quantitative methods built into the analysis pipeline and are described in the main text and the Method Details section. Power analysis to determine sample sizes was performed according to established methods (Charan and Kantharia, 2013; Festing and Altman, 2002) using OpenEpi software (http://openepi.com/SampleSize/SSPropor.htm). For differential transcriptome analysis, we used the statistical models built into the DESeq2 package, which fits read counts to a generalized linear model assuming a negative binomial distribution. We also compared RNA-seq data across species as well as with qRT-PCR data using Spearman’s rank-order correlation and multiple linear regression packages in Prism 8 software. Youden’s index J was calculated according to the standard formula: $J=\left(\left(\text{true}\;\text{positive}\;\text{rate}/\left(\text{true}\;\text{positive}\;\text{rate}\;+\;\text{false}\;\text{negative}\;\text{rate}\right)\right)\;+\;(\text{true}\;\text{negative}\;\text{rate}/(\text{true}\;\text{negative}\;\text{rate}+\text{false}\;\text{positive}\;\text{rate}))\right)\;-1$. Positive predictive values (PPV) were calculated using the sensitivity (true positive rate) and specificity (true negative rate) according to the formula: $\text{PPV}\;=\;\left(\text{sensitivity}\;x\;\text{prevalence}\right)\;/\left(\left(\text{sensitivity}\;x\;\text{prevalence}\right)+\left(1-\;\text{specificity}\right)+\left(1-\text{prevalence}\;\right)\right)$. Negative predictive values (NPV) were calculated using the sensitivity (true positive rate) and specificity (true negative rate) according to the formula: $\text{NPV}\;=\left(\text{specificity}\;+\left(1-\;\text{prevalence}\right)\right)/(\left(1-\;\text{sensitivity}\;\right)\times \;\text{prevalence}\;+\;\text{specificity}\times \left(1-\text{prevalence}\right)$.

#### Statistical significance

We defined significant differences in survival, body weight, or viral titer as those with an adjusted p value < 0.01. Differential gene expression was defined by fold-change relative to time-matched, mock-infected controls > |1.5| and an adjusted p value < 0.01 (for standalone transcriptomic analyses) and an adjusted p value < 0.05 (for integrated miRNA-mRNA analyses). Functional enrichment was determined by IPA Core Analysis by Fisher’s exact test with Benjamini-Hochberg false discovery rate correction.

Exact values of n are as follows: At least 5 mice per CC line per survival/weight experiment. 3 mice per condition (infected or time-matched mock-infected controls) per time point per CC line. Survival experiments were repeated to confirm reproducibility of phenotype.

## Supplementary Material

Supplemental Figures

Supplemental Sheet 1

Supplemental Sheet 2

Supplemental Sheet 3

Supplemental Sheet 4

Supplemental Sheet 5

## Figures and Tables

**KEY RESOURCES TABLE:** 

REAGENT or RESOURCE	SOURCE	IDENTIFIER

Antibodies		

Mouse monoclonal anti-VP40	Yoshihiro Kawaoka, University of Wisconsin-Madison	N/A
FITC goat anti-mouse secondary	MilliporeSigma	RRID: AB_259378; Catalog #F0257; MDL #MFCD00162642

Bacterial and Virus Strains		

Mouse-adapted Ebola virus	USAMRIID	GenBank: AF499101

Biological Samples		

Spleen and liver collected from Collaborative Cross mice	University of North Carolina Systems Genetics Core (RRID:SCR_016401)	N/A

Chemicals, Peptides, and Recombinant Proteins		

Trizol reagent	ThermoFisher	Catalog #15596018
RNAlater	ThermoFisher	Catalog #AM7021
Direct-zol MiniPrep RNA Extraction kit	Zymo Research	Catalog #R2071

Critical Commercial Assays		

SuperScript III Platinum One-Step RT-PCR Kit	ThermoFisher	Catalog #12574035
Bioanalyzer RNA 6000 Nano Kit	Agilent Technologies	Catalog #5067–1511
High Sensitivity ScreenTape	Agilent Technologies	Catalog #5067–5579
HiSeq 4000 sequencer	Illumina	RRID: SCR_016386
TruSeq RNA Library Prep Kit v2	Illumina	Catalog #RS-122–2001
TruSeq Small RNA Library Prep Kit	Illumina	Catalog #RS-200–0012

Deposited Data		

RNA-seq data from CC mouse spleen and liver	This study	NCBI GEO: GSE130629
RNA-seq data from Guinean EVD patients	Liu et al., 2017	NCBI BioProject: PRJNA352396

Experimental Models: Cell Lines		

Vero E6	American Type Culture Collection	RRID: CVCL_0574; ATCC #CRL-1587

Experimental Models: Organisms/Strains		

Collaborative Cross mice	UNC Systems Genetics Core (RRID:SCR_016401) David Threadgill, Texas A&M University	N/A

Oligonucleotides		

5’- GCAGAGCAAGGACTGATACA-3’ (Forward primer for Ebola virus GP)	IDT	N/A
5’- GTTCGCATCAAACGGAAAAT-3’ (Reverse primer for Ebola virus GP)	IDT	N/A
5’-FAM- CAACAGCTTGGCAATCAGTAGGACAT-TAMRA-3’ (Probe for Ebola virus GP TaqMan)	IDT	N/A

Software and Algorithms		

Ingenuity Pathway Analysis	QIAGEN Bioinformatics	RRID: SCR_008653
DEVis	Price et al., 2019	N/A
Trimmomatic	Bolger et al., 2014	RRID: SCR_011848
FastQC	http://www.bioinformatics.babraham.ac.uk/projects/fastqc/	RRID: SCR_014583
STAR	Dobin et al., 2013	RRID: SCR_015899
featureCounts	Liao et al., 2014	RRID: SCR_012919
DESeq2	Love et al., 2014	RRID: SCR_015687
DCQ/ComICS	Altboum et al., 2014	N/A
biomaRt	Yates et al., 2016	RRID: SCR_002987
XGSA	Djordjevic et al., 2016	N/A
OpenEpi	http://openepi.com/SampleSize/SSPropor.htm	N/A
Prism 8	GraphPad Software	RRID: SCR_002798
Cytoscape	Shannon et al., 2003	RRID: SCR_003032
ClueGO	Bindea et al., 2009	RRID: SCR005748


## Data Availability

The published article includes all datasets generated or analyzed in this study, excepting raw RNA-seq FASTQ data. All mouse-derived RNA-seq data performed for this study is available via the Gene Expression Omnibus (GEO) resource. The accession number for the raw sequence data is NCBI GEO: GSE130629. Additional RNA-seq data from EVD patients used in analyses is available for download from NCBI BioProject (NCBI BioProject: PRJNA352396). All code is open source and freely available as R or Python packages or via the open source Cytoscape platform, with the exception of IPA and Prism 8, which are proprietary products that require a paid license from QIAGEN Bioinformatics and GraphPad Software, respectively. All data or analytical results not included in the Supplemental Data are available on request from the corresponding author.

## Highlights

- Host responses produce distinct Ebola virus outcomes in genetically diverse mice
- Tolerant outcomes result from early inflammatory responses and sustained regulation
- Lethality is linked to early gene suppression and uncontrolled inflammation
- Murine transcriptional profiles can be used to predict clinical outcome in humans
