# Supplementary material for: Transcriptional Correlates of Tolerance and Lethality in Mice Predict Ebola Virus Disease Patient Outcomes
Source: Cell Rep. Author manuscript; Available in PMC 2024 May 1. (PMC11062563; doi:10.1016/j.celrep.2020.01.026)
Supplement: Supplemental Figures [file NIHMS1986033-supplement-Supplemental_Figures.pdf]

Cell Reports, Volume *30*

Supplemental Information

Transcriptional Correlates of Tolerance and Lethality in Mice Predict Ebola Virus Disease Patient Outcomes

Adam Price, Atsushi Okumura, Elaine Haddock, Friederike Feldmann, Kimberly Meade-White, Pryanka Sharma, Methinee Artami, W. Ian Lipkin, David W. Threadgill, Heinz Feldmann, and Angela L. Rasmussen

Table S1. CC Lines Used in Study. Related to STAR Methods.

CC Line	Disease Outcome	Mortality	Disease Phenotype
CC011/Unc	Tolerant	0%	Transient morbidity
CC021/Unc	Tolerant	0%	Transient morbidity
CC026/GeniUnc	Tolerant	0%	Transient morbidity, splenomegaly, hepatitis
CC041/TauUnc	Lethal	100%	Lethal with hemorrhagic syndrome
CC042/GeniUnc	Lethal	100%	Lethal
CC043/GeniUnc	Lethal	100%	Lethal with hemorrhagic syndrome
CC055/TauUnc	Lethal	100%	Lethal with hemorrhagic syndrome
CC057/Unc	Tolerant	20%	Transient morbidity, splenomegaly, hepatitis
CC061/GeniUnc	Tolerant	0%	Transient morbidity, splenomegaly
CC065/Unc	Tolerant	20%	Transient morbidity

Lethal with hemorrhagic syndrome refers to lethal disease with splenomegaly, hepatitis, and coagulopathy (prolonged aPTT/PTT, low serum fibrinogen, or evidence of disseminated intravascular coagulation and coagulopathy at necropsy)

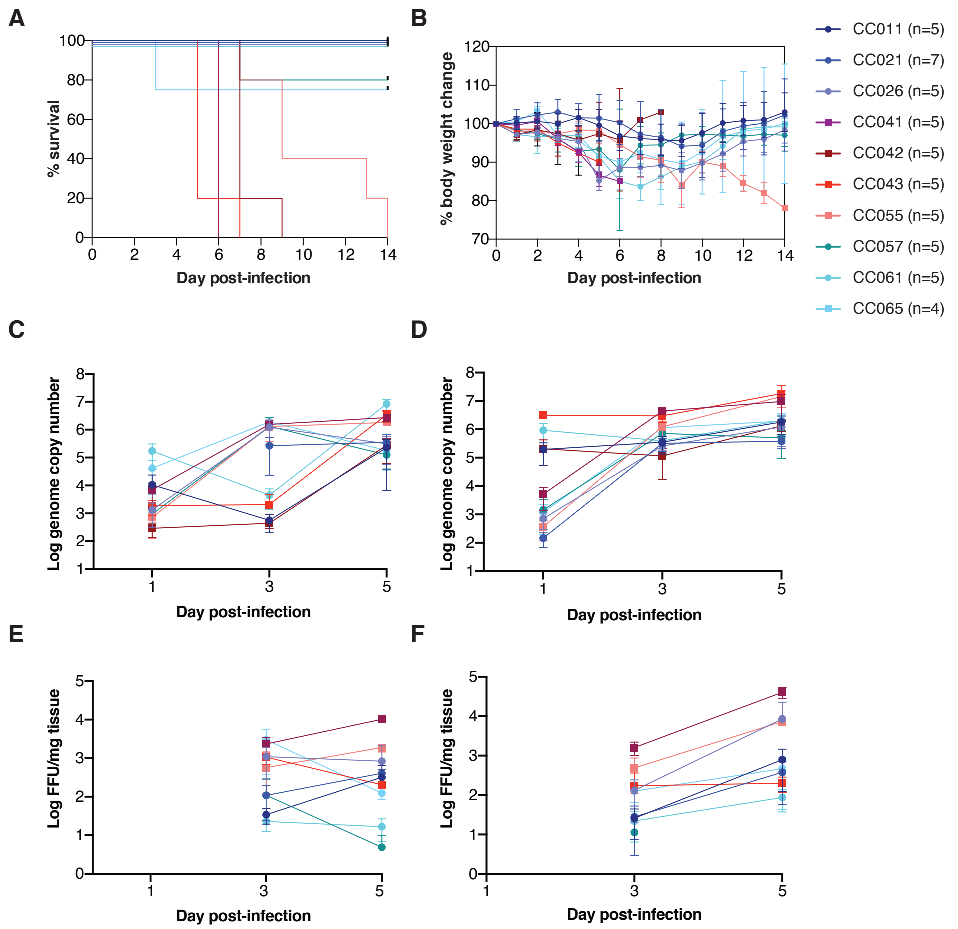

Figure S1. Clinical and virological data from individual CC lines. Related to Figure 1. (A) Kaplan-Meier survival curve in tolerant (CC011, indigo, 5 mice; CC021, cobalt, 7 mice; CC026, periwinkle, 5 mice; CC057, teal, 5 mice; CC061, turquoise, 5 mice; CC065, cerulean, 4 mice) versus lethal (CC041, magenta, 5 mice; CC042, maroon, 5 mice; CC043, red, 5 mice; CC055, salmon, 5 mice) mice over 14 days p.i. **(B)** Body weight percent change from the time of infection (day 0) recorded daily in mice used to assess mortality. Viral RNA (log_{10} genome copies) in spleen **(C)** and liver **(D)** in tolerant versus lethal mice at days 1, 3, and 5 p.i. (3 mice/CC line/time point). Infectious titers (log_{10} focus-forming units per mg tissue) in tolerant versus lethal mice at days 1, 3, and 5 p.i. (3 mice/CC line/time point).

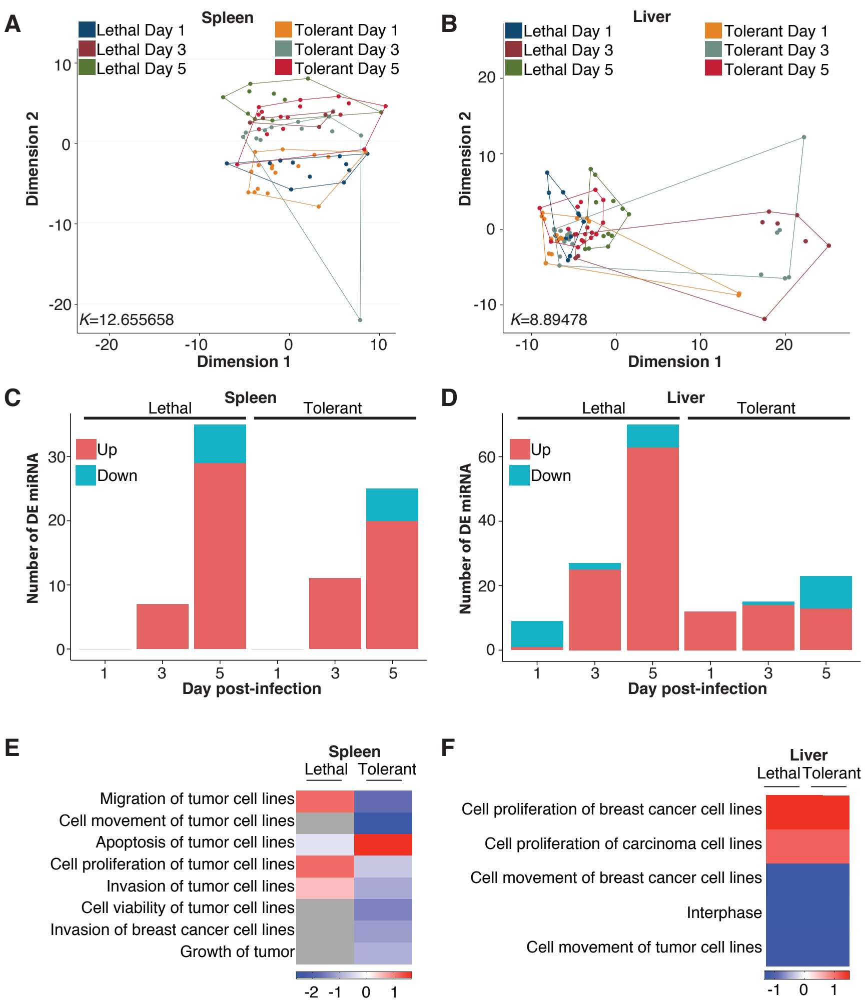

Figure S2. Global EVD outcome-dependent miRNA transcriptomic profiles. Related to Figure 3. Multi-dimensional scaling (MDS) of all differentially expressed (DE) miRNA in spleen **(A)** and in liver **(B)** by outcome and time. K-values are indicative of Kruskal's stress (formula 1), a measure of fit and information loss in dimensionality reduction. **(C, D)** Number of miRNA meeting DE criteria (fold change > |1.5|, adjusted p < 0.01) spleen **(C)** and liver **(D)**. Blue indicates down-regulation and red indicates up-regulation relative to time-matched, mock-infected controls. **(E,F)** Functional enrichment in spleen **(E)** and liver **(F)** determined by IPA Core Analysis. Pathway enrichment was determined by Fisher's exact test and *p*-values were adjusted using the Benjamini-Hochberg false discovery rate correction. All corrected enrichment *p*-values were < 0.01. Red indicates a positive z-score (pathway activation), blue indicates a negative z-score (pathway inhibition), white indicates that a z-score could not be calculated, and gray indicates no pathway enrichment. Transcriptomic data was generated using spleen and liver from mice and analyzed by outcome relative to time-matched mock-infected controls from each line (3 mice/time point/condition/line at 3 time points). We used miRNA transcriptomic data from tissues collected in 6 tolerant lines 6 tolerant lines (54 infected mice and 54 time-matched mock-infected controls) and 4 lethal lines (36 infected mice and 36 time-matched mock-infected controls).

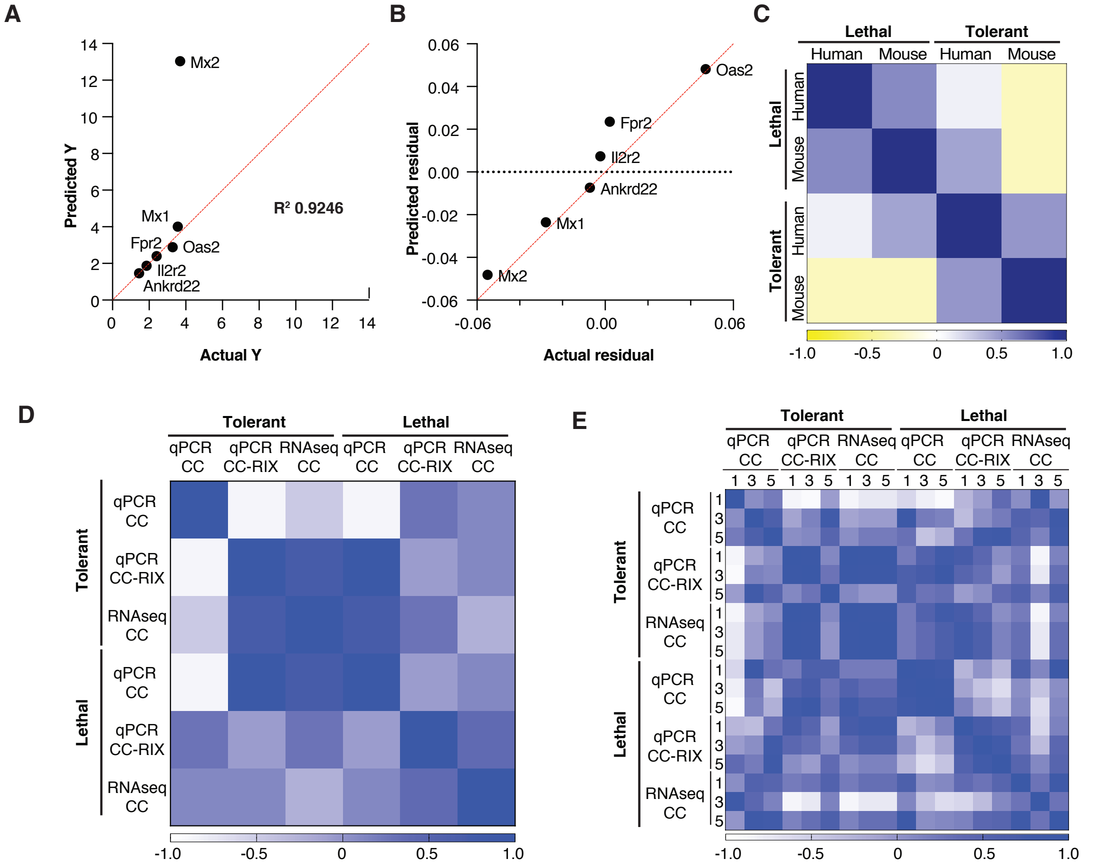

Figure S3. Correlation of classifier gene expression across species and platforms. Related to Figure 5. (**A**) Multiple linear regression plot of spleen and human classifier gene expression. (**B**) Q-Q plot of linear regression residual distribution. (**C**) Heatmap showing Spearman's rank-order correlation between mouse and human classifier gene expression tested by EVD outcome. Blue shading indicates positive correlation, yellow shading indicates negative correlation. (**D,E**) Heatmaps showing Spearman's rank-order correlation between mouse classifier gene expression by RNAseq and qRT-PCR by EVD outcome at all time points (**D**) and each time point (**E**). Correlation analysis was performed on a 6-gene classifier subset of RNAseq DE data and qRT-PCR data from spleen tissue from 60 male CC mice from the 10 CC backgrounds used in this study and an independent set of spleen RNA collected from 36 male CC-RIX mice from 6 CC-RIX backgrounds (3 mice per time point per group). Blue shading indicates positive correlation, white shading indicates negative correlation.

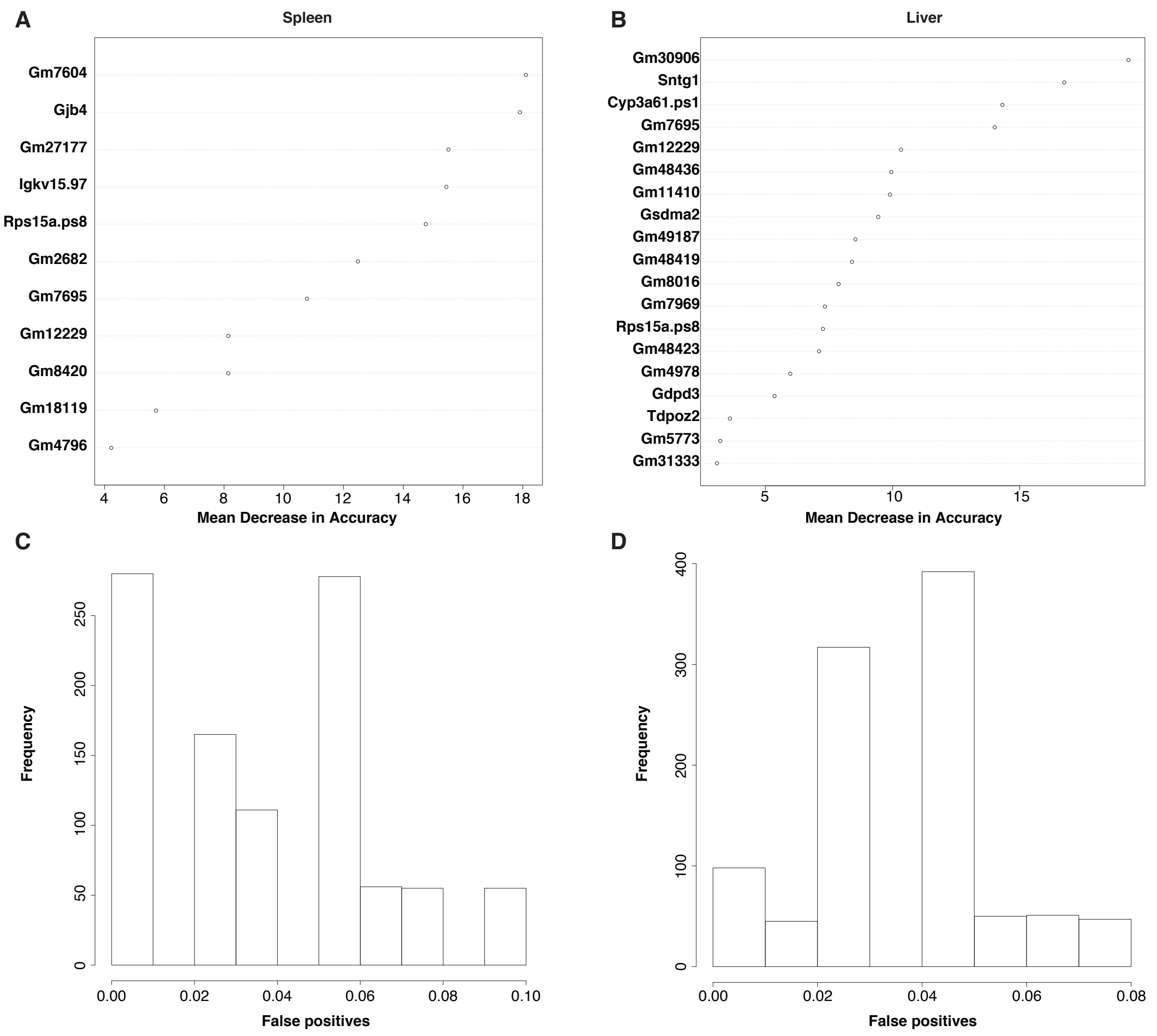

Figure S4. Mouse classifier performance at all time points in spleen and liver. Related to Figure 5. Mean decreases in accuracy of mouse spleen **(A)** and liver **(B)** gene profiles used for RF prediction of outcome. Histograms showing the frequency of false positives across all 1000 bootstrapped RF runs in spleen **(C)** and liver **(D)**.

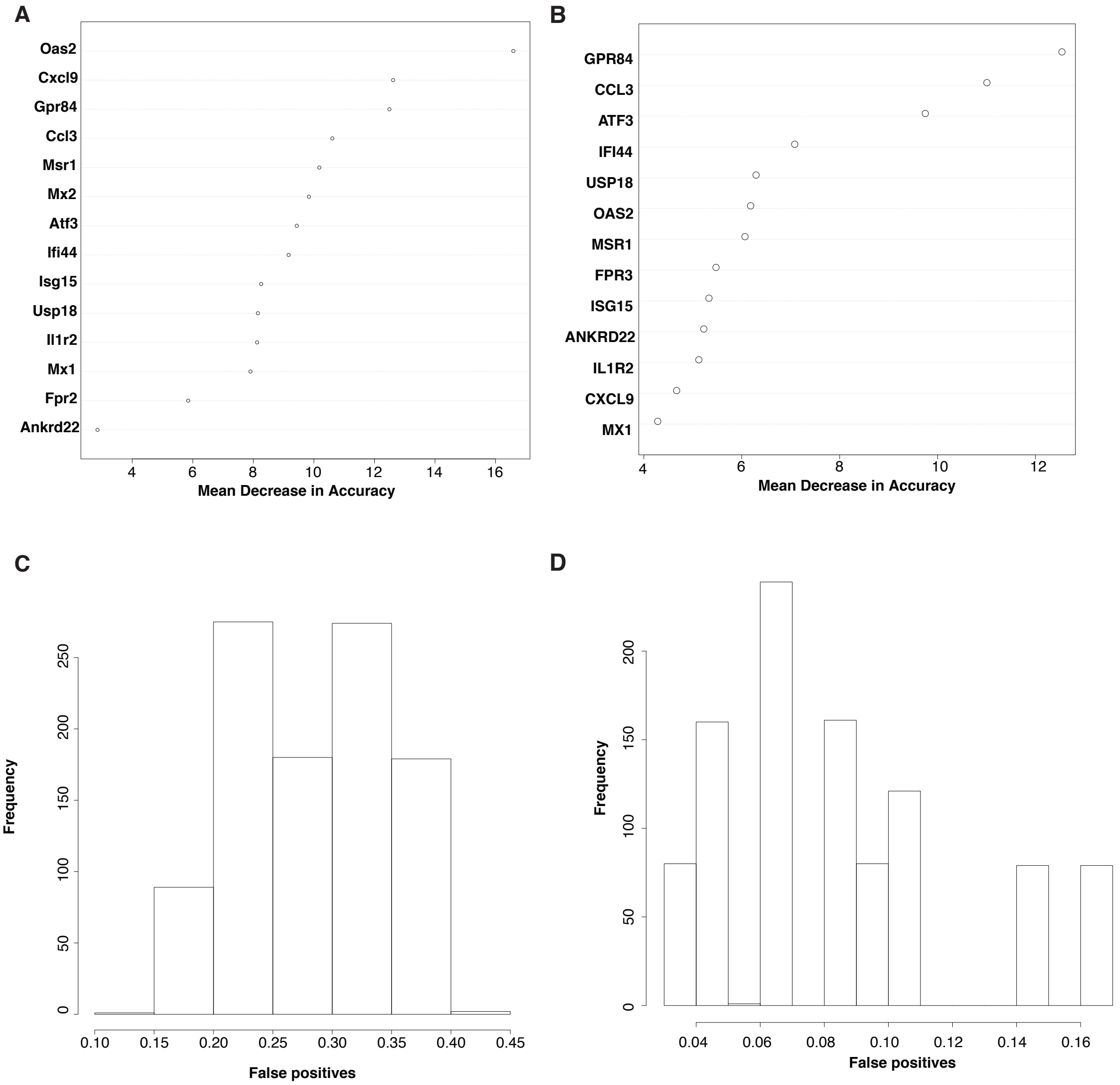

Figure S5. Cross-species classifier performance at all time points tested in mouse spleen and human peripheral blood. Related to Figure 5. (A,B) Mean decrease in accuracy of orthologous profile used for RF prediction of outcome in mice (**A**) and humans (**B**). **(C,D)** Histogram showing the frequency of false positives across all 1000 bootstrapped RF runs in mice **(C)** and humans **(D).**

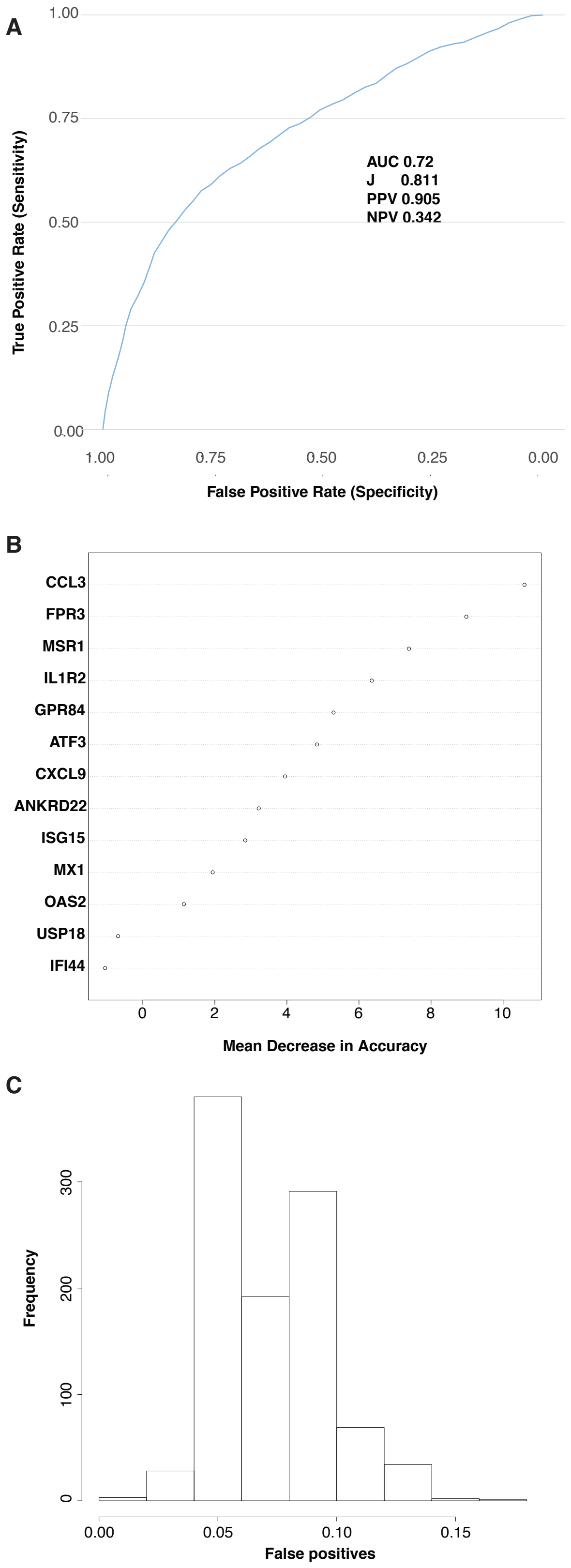

Figure S6. Cross-species classifier performance in unfiltered human sample set. Related to Figure 5. (A) ROC curve showing sensitivity and specificity of outcome prediction of orthologous classifier tested using the full data set of all human EVD samples. **(B)** Mean decrease in accuracy of orthologous profile used for RF prediction of outcome **(C)** Histogram showing the frequency of false positives across all 1000 bootstrapped RF runs.
